# Maternal, Sexual and Reproductive Health in Marginalised Areas: Renewing Community Involvement Strategies beyond the Worst of the COVID-19 Pandemic

**DOI:** 10.3390/ijerph19063431

**Published:** 2022-03-14

**Authors:** Grant Murewanhema, Godfrey Musuka, Chipo Gwanzura, Richard Makurumidze, Itai Chitungo, Munashe Chimene, Nigel Tungwarara, Tafadzwa Dzinamarira, Mugove Gerald Madziyire

**Affiliations:** 1 Unit of Obstetrics and Gynaecology, Department of Primary Health Care Sciences, Faculty of Medicine and Health Sciences, University of Zimbabwe, Harare P.O. Box MP167, Zimbabwe; gmurewanhema@gmail.com (G.M.); chipo.gwanzura@gmail.com (C.G.); mmadziyire@medsch.uz.ac.zw (M.G.M.); 2 ICAP at Columbia University, Harare P.O. Box MP167, Zimbabwe; gm2660@cumc.columbia.edu; 3 Unit of Community Medicine, Faculty of Medicine and Health Sciences, University of Zimbabwe, Harare P.O. Box MP167, Zimbabwe; rmakurumidze@medsch.uz.ac.zw; 4 Chemical Pathology Unit, Faculty of Medicine and Health Sciences, University of Zimbabwe, Harare P.O. Box MP167, Zimbabwe; ichitungo@medsch.uz.ac.zw; 5 Department of Health Sciences, Africa University, Mutare P.O. Box 1320, Zimbabwe; chimenem@africau.edu; 6 Department of Health Studies, University of South Africa, Pretoria 0002, South Africa; 34846751@mylife.unisa.ac.za; 7 School of Health Systems & Public Health, University of Pretoria, Pretoria 0002, South Africa

**Keywords:** COVID-19, pandemic, family planning, antenatal care, postnatal care, sexually transmitted infections, sexual and gender-based violence

## Abstract

The COVID-19 pandemic and resultant lockdowns have brought unprecedented challenges for Maternal, Sexual and Reproductive Health (MSRH) services. Components of MSRH services adversely affected include antenatal, postnatal, and newborn care; provision of family planning and post-abortion care services; sexual and gender-based violence care and prevention; and care and treatment for sexually transmitted infections including HIV. Resuscitating, remodeling or inventing interventions to restore or maintain these essential services at the community level, as a gateway to higher care, is critical to mitigating short and long-term effects of the COVID-19 pandemic on essential MSRH. We propose a possible framework for community involvement and propose integrating key information, education, and communication of MSRH messages within COVID-19 messages.

## 1. Introduction

The severe acute respiratory syndrome coronavirus 2 (SARS-CoV-2), the causative agent of coronavirus disease 2019 (COVID-19), was first detected in the Chinese Wuhan City in December 2020, and rapidly evolved into a global pandemic by March 2020 [1]. Countries around the world put in place different measures to curb importing the virus from other countries and to contain in-country transmission. The measures were of different severities across the countries; however, they almost universally affected different aspects of people’s lives. One key aspect that the World Health Organization (WHO) encouraged governments to ensure was the continuity of essential health services [2]. Other countries, such as Zimbabwe, initially imposed a full-scale lockdown on their populations, preventing people from accessing critical health services [3,4,5]. 

The COVID-19 pandemic has significantly evolved over the past two years, with most countries having gone through 3–4 waves, driven by different variants of concern, including the Alpha, Beta, Delta, and more recently, the Omicron variant [6]. As the pandemic has become more protracted than initially anticipated, with the possibility of the continued emergence of further waves, countries have to start thinking about living with the virus for an unforeseeable future, especially as vaccines have provided an important preventive tool, and the clinical disease picture has changed significantly. The disease attributable to the Omicron variant was substantially more attenuated than the Beta and Delta variants. This is partly attributable to a highly transmissible yet less virulent variant, and to the effectiveness of vaccination.

At the onset of the pandemic in March 2020, the Zimbabwean government introduced lockdown and movement restrictions to contain the COVID-19 pandemic, prepare for increased caseload and reduce the adverse health and socioeconomic consequences associated with the further spread of SARS-CoV-2. These measures may have negative impacts on healthcare delivery and access for women and girls [7]. Previous outbreaks such as the Ebola virus disease outbreak in Sierra Leonne and the Democratic Republic of Congo provided valuable insights on the consequences of humanitarian disasters and consequent public health interventions on Maternal, Sexual and Reproductive Health (MSRH) [8,9]. The United Nations postulates that women and girls are more likely to suffer from sexual and gender-based violence, and their reproductive rights are likely to remain unmet due to the prioritisation of government expenditure towards the fight against the pandemic [10].

A proactive approach is critical to mitigating adverse sequelae on MSRH during the COVID-19 pandemic and remaining on track to achieve Sustainable Development Goal number 3 (SDG3). SDG3 encompasses essential elements of MSRH, which include reducing the global maternal mortality ratio to less than 70 per 100,000 live births and ensuring universal access to sexual and reproductive health services, including family planning by 2030 [11]. In this viewpoint, as the COVID-19 has become protracted and countries are looking at ways of living with the virus, we discuss how community participation can be a valuable complement to mainstream healthcare services in mitigating against the negative consequences of the pandemic on essential MSRH services in Zimbabwean societies. Models that involve communities must be economical, sustainable and robust, given that the majority of rural Zimbabweans are poor and struggled with accessing healthcare services even before the pandemic. Therefore, frameworks that will work even beyond the worst of the COVID-19 pandemic are needed.

## 2. Community Participation in Primary Health Care

Community participation may be a valuable complement to conventional health initiatives. The Alma Ata declaration of 1978 recognised community participation as a critical component for the success of Primary Health Care [12]. Global health stakeholders now promote community participation as a vital component of a human rights-based approach to health [13]. A community refers to a group of individuals, families or other networks and social circles that provide support and are often the unit on which health activities are organised and focused. The involvement of communities, use of indigenous resources and reliance on peer support may improve health outcomes. Critical to the success of community involvement is the addressing of the social determinants of health and issues around power and control over decisions regarding community health and behaviour change.

### Communities and Some Critical Maternal, Sexual and Reproductive Health Interventions in Low and Middle-Income Countries

Communities have been at the centre of success for some MSRH interventions in low and middle-income countries (LMICs). The literature suggests that the empowerment of traditional birth attendants may reduce adverse maternal outcomes in guided contexts, primarily through health promotion and facilitating health facility use [14]. These findings were noted in settings such as Burundi [14], Sierra Leonne [15] and Guatemala [16]. A systematic review and meta-analysis of cluster randomized controlled trials in comparable low-resource settings demonstrated that the training of the traditional birth attendants can lead to reduced maternal and perinatal mortality [17]. In a feasibility study in Zimbabwe, it was noted that the scope of health promotion activities carried out by traditional birth attendants could be expanded to HIV prevention of mother-to-child transmission services [18]. Community Art Refill Groups have been instrumental in the continuity of highly-active antiretroviral therapy (HAART) uptake in some communities such as in Thyolo in Malawi, ensuring adherence and continued viral suppression [19]. In Zimbabwe, the Medecins sans Frontieres implemented this concept successfully in some districts, eventually leading to the incorporation of the concept into the Ministry of Health and Child Care’s differentiated models of HIV care [20].

In an integrative review that retrieved studies from Afghanistan, Bangladesh, Ethiopia, Gambia, Ghana, Kenya, Mozambique, Zambia and others, the community distribution of misoprostol tablets by community health workers led to reduced adverse outcomes from post-partum haemorrhage in these resource-limited countries [21]. In strategies to increase post-abortion care services in Angola, community nurses can successfully manage miscarriages with misoprostol at the community level, reducing referrals to health facilities [22]. The empowerment of women’s groups has resulted in increased contraceptive uptake in some marginalised communities in sub-Saharan Africa [23]. These examples attest to the fact that delivering MSRH services to women at a community level can lead to significantly improved outcomes. Central to success is giving the women a sense of ownership/belonging to these programmes as the intended beneficiaries.

## 3. Core Aspects of Maternal, Sexual and Reproductive Health

The core aspects of MSRH include antenatal, perinatal, postpartum and newborn care, family planning (including infertility services), sexual and gender-based violence care and eliminating unsafe abortion and sexually transmitted infections including HIV and reproductive tract infections. During unprecedented times such as the COVID-19 pandemic, innovative ways of involving the community in reducing adverse MSRH consequences are critical. Some interventions may not necessarily be novel but need resuscitation or re-modelling in the wake of current challenges. We propose a framework for involving the community in closing gaps and mitigating adverse MSRH outcomes. We envisage that these interventions will be sustainable even beyond the COVID-19 pandemic. Critical to the success of community participation is adequate information, education and communication (IEC). Therefore, the risk communication and community engagement pillar of the Ministry of Health and Child Care of Zimbabwe that oversees the production and distribution of COVID-19 related IEC material, therefore, is central to success. Indeed, close coordination between the several pillars of the COVID-19 response in the Ministry of Health and Child Care such as surveillance, infection prevention and control, risk communication and community engagement and others is very important.

### 3.1. Provision of Family Planning Services and Post-Abortion Care

The United Nations Population Fund (UNFPA) estimates the average national unmet need for contraception in Zimbabwe to be 10.4% and is considerably higher in the 15–19 years age group at 12.6% [24]. COVID-19 travel restrictions, supply chain disruptions and reprioritisation of expenditure are disrupting access to family planning services for women in the country. Whilst we have no specific estimates for Zimbabwe, global estimates suggest an additional 15 million unintended pregnancies with 28,000 maternal and 168,000 newborn deaths for a 10% drop in contraceptive service coverage [25]. Likewise, a 10% reduction in access to safe abortions in 1 year could increase unsafe abortions by an additional 3.3 million resulting in an additional one thousand maternal deaths [25].

The most popular methods of family planning used by Zimbabwean women include oral contraceptive pills, the injectable depo medroxyprogesterone acetate and the subdermal implant Jadelle. The uptake of other long-acting reversible contraception methods such as the copper intrauterine devices is low in Zimbabwe, and so is the uptake of permanent methods, which are tubal ligation for females and vasectomy for males. In fact, due to the patriarchal nature of Zimbabwean societies, family planning is usually relegated to women. Therefore, males are rarely involved. With very little support from the government, international development partners usually support the provision of contraceptives in the country. Other partners sometimes carry out community outreaches to encourage the uptake and provision of long-acting reversible contraceptives. Due to the pandemic’s requirements for infection prevention and control, these services were halted.

Gaps exist in access to family planning services, especially in the marginalised communities of Zimbabwe that could suffer disproportionately, owing to COVID-19 containment interventions including the unavailability of transport and closure of health facilities. Local and international partners who regularly support the provision of family planning services may have temporarily shifted attention to COVID-19 control. In Table 1, we propose some interventions to ensure continued access, especially in marginalised communities.

Access to safe post-abortion care in time is important for reducing the morbidity and mortality associated with these abortions. Abortions, especially the unsafe ones, conducted under unsterile and dangerous conditions can substantially increase associated adverse outcomes through post-abortal haemorrhage, sepsis and trauma such as that which occurs when penetrating objects are used to terminate pregnancies [26]. Timely access to post-abortion care allows access to antibiotics where needed, evacuations of the uterus which aid in stopping bleeding and infections and the identification of injuries to other structures such as the bowel. In the context of marginalised areas of Zimbabwe, these services are usually available at district hospitals and mission hospitals, and therefore the population requires public transport to reach these centres. Due to restrictions in transport to control the spread of SARS-CoV-2, delays are occurring in the decisions to seek care and reach health facilities once the decision to seek care has been made [3,4,5,27].

### 3.2. Antenatal and Postnatal Care Services

Focused antenatal care services were disrupted, resulting in unsatisfactory care and surveillance for pregnant women. Pregnant women are therefore not receiving micronutrient supplements, antenatal HIV testing and treatment services, tetanus toxoid and essential antenatal education. The burden of iron deficiency anaemia in rural communities due to reduced nutrient intake may be higher compared to urban communities [28]. The World Health Organization emphasized the need for good nutrition during the COVID-19 pandemic to maintain strong immunity, and this is even more important for pregnant women, hence the need to ensure that they have continued access to micronutrient supplements, even in times of adversities. The disruption of intermittent presumptive therapy for malaria may result in an upsurge of malaria in pregnancy, with adverse feto-maternal sequelae.

There is reduced utilisation of maternity services and a trend towards increased maternal and perinatal mortality [5,29]. Closer surveillance of the situation is warranted to reflect the true burden. Meanwhile, mitigating interventions are urgently required. Those involved in the care of newborn babies must find ways of reducing neonatal sepsis and complications and ensuring BCG vaccination for babies born in the community. We propose some possible interventions for the continuity of antenatal and postnatal care services at the community level during the COVID-19 pandemic in Table 2.

### 3.3. Sexually Transmitted Infections and HIV Care and Treatment Services

Statistical modelling suggests that a 6-month interruption in the provision of HAART could result in an excess of 500,000 deaths in Sub-Saharan Africa in 4 years, and double the vertical transmission of HIV [30]. UNAIDS estimates show that approximately 1.3 million people are living with HIV/AIDS in Zimbabwe, 67% of whom are women [30]. With effective treatment regimens, many are now falling pregnant, and another substantial proportion falls pregnant without knowledge of their HIV status or seroconvert in pregnancy. Failure to access HIV Care and Treatment services serves to reverse the Prevention-of-Mother-to-Child-Transmission (PMTCT) gains realised over the previous years. Differentiated models to ensure continuity of antiretroviral therapy provision especially for pregnant women and breastfeeding mothers are required urgently. We make suggestions for ensuring continued access to sexually transmitted infection prevention services and HAART at the community level during the COVID-19 pandemic in Table 3.

### 3.4. Sexual and Gender-Based Violence Care

Evidence from previous disasters suggests that women and girls are at a heightened risk of domestic violence, intimate partner violence, child abuse and some other forms of sexual and gender-based violence [9,31]. Disaster times exaggerate pre-existing societal power structures and gender inequities [31]. In Sierra Leonne, forces looking after quarantine centres reported sexually abused young girls during the Ebola outbreaks [31]. Coupled with the lack of timely access to care, women and girls may suffer from unintended pregnancies, sexually transmitted infections including HIV and prolonged post-traumatic stress disorder. Lack of access to basic MSRH services resulted in a 75% increase in maternal mortality in Guinea, Liberia and Sierra Leone as unintended pregnancies increased during the Ebola outbreak [9]. Interventions at the community level are critical to mitigate the gap between event and professional care, and suggestions are made in Table 4.

## 4. Community Interventions as a Gateway to the Formal Health Sector

It is important that community MSRH services do not become a substitute for formal healthcare services. Instead, they can be an important intervention for the identification of complications and challenges that require a timely referral, assessment and interventions at district, mission and other hospitals. In the past, community healthcare workers were very effective at treating simple malaria and referring those with severe malaria to hospitals for admission. Similarly, they can be trained to triage pregnant women and refer pregnancies deemed as high risk to health facilities in good time to avert potential complications.

### 4.1. Vaccination for Women of Reproductive Age and Community Health Workers

Like the rest of the world, Zimbabwe commenced COVID-19 vaccination for the adult population in February 2021 [32]. COVID-19 vaccination is one of the best public health interventions for the control of the pandemic. Vaccines reduce incident SARS-CoV-2 infections, reduce disease severity, limit hospitalisations and reduce deaths from COVID-19. As MSRH services are extended into communities, it is critical to make sure that both care providers (community health workers) and their clients, who are mainly women of reproductive age in the context of MSRH, are vaccinated. Unfortunately, there are no clear guidelines for the vaccination of pregnant women in Zimbabwe [33], and vaccine hesitancy among WRA is relatively higher compared to the rest of the population. This is because this population has safety concerns regarding COVID-19 vaccination, including fears of subfertility, miscarriage, adverse pregnancy outcomes and long-term development of their offspring [34]. Therefore, fighting vaccine hesitancy by appropriately addressing this population’s concerns is as important as availing the vaccines. Evidence of the safety of SARS-CoV-2 vaccines in this population must be availed and communicated during community outreach programmes [35].

Optimising the uptake of SARS-CoV-2 vaccines is important towards propelling countries to high proportions of vaccinated cohorts, reducing the chances of breakthrough infections and the continued emergence of variants of concern [36]. We, therefore, suggest that the government of Zimbabwe must develop full COVID-19 vaccination guidelines for the population of pregnant women, following the examples that have been set by other bodies such as the WHO, the Royal College of Obstetrics and Gynaecology (RCOG) and the American Congress of Obstetrics and Gynaecology (ACOG).

### 4.2. COVID-19 Information, Education and Communication

The success of public health interventions critically depends on adequate IEC. The COVID-19 risk communication and community engagement pillars in the various districts must engage with their communities to design appropriate IEC material to distribute within the communities, ideally in local languages. Key MSRH messages can be integrated within COVID-19 IEC material to improve efficiency. Local leaders, including parliamentarians, councillors and traditional leaders play an essential role in educating their communities. They must work closely with healthcare practitioners and educators in their areas to ensure that correct messages reach recipients. It is critical to dispel rumours, myths and misconceptions that can negatively influence key public health interventions such as vaccination. Ideal places for placing IEC material include community boreholes, shops and schools. Prevention of COVID-19 and other communicable diseases remains critical as communities move to provide some MSRH services. If not correctly implemented, well-meant interventions can cultivate disaster. Thus, community kiosks, for example, can end up as hotspots for SARS-CoV-2 transmission.

The risk communication and community engagement pillar has a central role in communicating COVID-19 vaccination messages, fighting vaccine hesitancy, falsehoods, rumours, myths and misconceptions and boosting vaccine confidence, thereby improving uptake of vaccines.

## 5. Critical Issues to Consider for Successful Implementation of Community MSRH Services

The success of community interventions is dependent on motivation and community participation. Involving communities right from the planning stages of the MSRH interventions and in assessing their own needs and developing strategies to meet those needs can increase intervention ownership and sustainability [37]. This will also help in determining an appropriate and realistic scope of work that addresses the priority MSRH service gaps in the community. It is important to consider the key community stakeholders around MSRH and this can include traditional birth attendants, religious leaders, and the community at large as this forges concessions and mutual understanding between the community and formal health systems. Non-financial performance-based incentives can be effective at increasing community health workers’ motivation and performance especially when the incentives promote social recognition. While financial performance-based incentives alone can improve service delivery outcomes, they pose a risk of unincentivized tasks being neglected [38,39]. This calls for a joint incentives approach by the organizations that run programs within the communities to avoid this negligence of other programs. This can be achieved if organizations supporting health programs can collaborate in creating a pooled fund for incentives with common incentivized service delivery outcomes such that the recipients of the incentives will not be biased towards programs, they perceive to be more financially rewarding. The COVID-19 pandemic has led to disruption of the workflow upon which community health workers rely; therefore, resources earmarked for performance-based incentives should be reallocated to cover routine salaries and stipends. For instance, village health workers in Zimbabwe’s Chipinge district are relying on the allowances paid through UNICEF for their livelihoods as earnings from farming and other activities were disrupted by the COVID-19 outbreak [40]. Community health workers must receive allowances for their time and commitment. Engaging key local partners and community stakeholders to support these interventions is therefore necessary.

Continuous education to minimise COVID-19 infection within the communities and among the community health workers who provide MSRH services is critical. Thus, basics of infection prevention and control, including hand-washing ports, alcohol-based sanitisers, facemasks and latex gloves need to be supplied. The provision of personal protective equipment is critical as the community health workers bridge the shortages of health workers which are more pronounced in rural areas [41]. The success of VHWs and CHWs in managing the expectations of the communities they serve will depend on the type and quality of support from the formal health system and from social networks within the communities. This support can further be compromised by the limited access to facilities because of distance, difficult terrain and lack of funds for transport [37,42].

Whilst telemedicine has been shown to be a useful intervention for managing women in obstetrics and gynaecology [43,44], internet and voice call charges could be a barrier to utilisation. Communities must engage with mobile service providers for subsidised charges or free access as corporate social responsibility. Community MSRH should aim to promote cultural norms of collective responsibility that remove the perception of women’s health as the responsibility of the individual. The communities where this shift in norms occurs are more likely to sustain their efforts to improve health and maintain mechanisms such as transport systems compared with those that remain focused on individual responsibility [37]. Furthermore, inclusion and support of men as equal partners can improve sexual and reproductive health indicators, such as contraception acceptance, safer sexual behaviours, use of reproductive health services and reduction in SRH related morbidity and mortality [45,46].

It is important to develop capacity-building strategies for the communities in relation to MSRH knowledge, skills and abilities. The training contents should be adapted to local needs, using appropriate training methodologies for adult learning with considerations of training material being dispensed in local languages. The trained community cadres will also require additional training, refresher courses and regular supportive supervision which can be provided through the various stakeholders operating in these communities.

Like any other community health intervention, there is a need for research support to measure the effectiveness of the interventions and evaluate the implementation itself with an aim of learning lessons for national and regional scale-up. Stakeholder collaborations that involve the government, non-governmental organisations and academic institutions will help in developing evidence-based interventions. Findings from such evaluations will also help in making communities appreciate the health benefits of the programs thereby enhancing participation and ownership.

## 6. Conclusions

As health facilities remain operating sub-optimally, innovative ways of ensuring continued access to essential MSRH services at the community level are needed. Active community participation and delivery of services at the community level can reduce the undesirable consequences that stem from a failure to access services. Thus, communities and key stakeholders must work closely together to ensure continuity. However, community interventions must be used as a gateway to the formal health sector, and women requiring medical attention must be urgently referred to, to prevent avoidable morbidity and mortality. The government in the meanwhile must find ways of urgently restoring essential health services in accordance with technical guidance from the WHO. Optimising vaccination for community health workers and women of reproductive age including pregnant women is important for ensuring the safety of these populations, including reduced adverse outcomes from severe COVID-19. The government must therefore finalise guidelines for pregnant women and adequately fight vaccine hesitancy adequately among women of reproductive age.

## Figures and Tables

**Table 1:** Maintaining essential family planning activities and managing abortions at the community level during the COVID-19 pandemic.

Item and Objective	Action Points
Train village health workers: to ensure continuity of access to essential family planning services	Village health workers can be trained to distribute oral contraceptives in the community, to reduce travel requirements and bring contraceptives closer to where the clients stay; Provide village health workers with tools for infection prevention and control to protect themselves and their community whilst they distribute contraceptives.
Community Kiosks: to ensure easy access of family planning methods at strategic points in communities	Women can access contraceptive pills and condoms at community kiosks manned by village health workers or primary care nurses stationed in villages, to reduce the need to travel; Place IEC material at these community kiosks and aim to integrate family planning and other MSRH messages within key COVID-19 IEC messages, incorporating colourful displays.
Provide long-acting reversible contraception via mobile clinics: to reduce the need for repeated visits to health centres	Long-acting reversible methods of contraception such as Jadelle, Implanon and Copper Intra-uterine contraceptive devices can be offered via mobile clinics observing minimum aseptic techniques; Village health workers, village heads and other community leaders can spread information regarding visit schedules.
Establish telemedicine services: to minimize person-to-person contact with healthcare providers in avoidable circumstances	Provide communities with linkage to telemedicine services in case they encounter adverse effects to contraception. This enables triaging to determine who needs attention in a medical facility: ○ With government medical officers ○ With gynaecologists
Managing abortion in the community: to reduce adverse outcomes associated with reduced access to post-abortion care	Community nurses can work with community health workers for the administration of misoprostol to stable patients awaiting access to safe health facilities. Liaising with midwives and medical officers through teleconsultations will be critical; Community health workers must be empowered to identify and facilitate the urgent referral of all unstable patients to health facilities.

**Table 2:** Ensuring the continuity of essential antenatal and postnatal care services at the community level during the COVID-19 pandemic.

Item and Objective	Action
Provide micronutrient supplements and malaria prophylaxis tablets at the community level: to reduce risk of anaemia and malnutrition, and reduce adverse outcomes from malaria in pregnancy in endemic areas	Village health workers, village heads and other community leaders can keep a diary of all pregnant women in their locality; Village health workers to provide all pregnant women in their communities with micronutrient supplements and provide them with three-month supplies of iron and folate; Village health workers can be educated on dosing schedules for intermittent presumptive therapy of malaria with sulphadoxine-pyrimethamine and provide correct doses to pregnant women in malaria-endemic areas.
Establish mobile antenatal care clinics and mobility for community nurses: to ensure continued access to essential antenatal care and surveillance in marginalized communities	Mobile antenatal clinics. These can be integrated with mobile family planning and HIV care services, and the same basic precautionary infection prevention and control measures taken as above; Village heads, community leaders and village health workers can spread dates of scheduled visits into the community; Ministry of Health and Child Care and development partners to support the mobile clinics with basic personal protective equipment, sphygmomanometers, thermos-scanners and hand-held dopplers; Ministry of Health and Child Care and development partners can provide motorbikes for community nurses and community health workers, with thermoscanners and blood pressure machines, to monitor pregnant and postpartum patients; Advocate for observation of physical distancing and attending to limited numbers of clients during these visits. A strict booking register can be observed; Any clients with high-risk pregnancies should be referred to higher facilities.
Community distribution of misoprostol: to reduce adverse outcomes from postpartum haemorrhage for women who face challenges in accessing health centres in time	Village health workers are provided with misoprostol to distribute to pregnant women near term in case they fail to access health facilities when they get into labour due to transport challenges.
Transport and logistics for labouring women: to reduce the logistical challenges in accessing transport and minimize delays in reaching health centres for labour and delivery	Villages/Communities must ideally locate a resident with a reliable vehicle, and pool funds to ensure it is always fuelled; This individual(s) can assist with getting pregnant women to nearby health facilities when they get into labour, and they must be given exemption letters to allow them to travel freely; Sensitise local leaders (chiefs, headmen) on the importance of timely access to healthcare facilities for labouring women. Most chiefs are provided with government vehicles that can be used to assist pregnant women in marginalised areas.
Postnatal care: to ensure continued access to essential postnatal care services at the community level	Village health workers and other community health workers can be sensitised to follow-up pregnant women who cannot access health facilities, to identify any dangerous symptoms and signs that need urgent intervention; Smooth communication between midwives, medical officers and local leadership can facilitate urgent referral of such women to higher facilities.

**Table 3:** Ensuring continued access to sexually transmitted infection prevention and HAART services at the community level during the COVID-19 pandemic.

Item and Objective	Action
Community antiretroviral therapy delivery models for pregnant and breastfeeding women: to ensure continued access to antiretroviral therapy with no disruptions	Development partners in conjunction with the Ministry of Health and Child Care to urgently design differentiated models of antiretroviral therapy delivery for pregnant women and breastfeeding mothers; All pregnant women must be granted exemption letters to enable them free movement between village and health facility levels. At the community level, village heads and chiefs can be granted this authority; Ensure maximumly permissible (3–6 months) supplies of HAART and Co-trimoxazole are given for each client; Groups can apply to partners for funding for transport allowances for the appointed members.
Mobile antiretroviral therapy clinics: to ensure continued access to HIV treatment in the communities, closer to where people stay	Pregnant women must be given priority for testing; Ensure test and treat strategy is employed effectively for any pregnant mothers who test positive for HIV to minimise vertical transmission; Appropriate counselling services must be maintained for women to ensure adherence and compliance to medicines; Village health workers, community leaders and village heads can spread the news of scheduled dates.
Telemedicine: to minimize unnecessary face-to-face contacts between clients and service providers	Establish links for telemedicine with medical officers and specialist doctors for side-effects and other problems; Communities to advocate for phones and subsidised calls to ensure availability of these services
Sexually transmitted infections prevention at the community level: to ensure continued access to these preventive services for those in need	Condom distribution at community kiosks; Condom distribution through village health workers and other community health workers for free; Reinforce messages on the importance of safer sex and abstinence at key and strategic points in the community; Integrate COVID-19 prevention messages with messages on HIV and sexually transmitted infections prevention.

**Table 4:** Ensuring early care for sexual and gender-based violence and rape survivors at the community level.

Item	Action
Disclosure of sexual and gender-based violence: to ensure timely access to care and treatment for victims	Primary care counsellors and community health workers must be empowered to encourage early disclosure of sexual and gender-based violence by victims to promote early seeking of appropriate treatment and counselling services.
Emergency Contraception: to reduce the risks of unwanted pregnancies for rape survivors	Village health workers and community health workers must be trained to provide emergency oral contraception to survivors at the community level within 12–72 h to prevent unintended pregnancies.
Post-Exposure Prophylaxis: to reduce the risk of incident HIV and sexually transmitted infections from rape incidents	Provide post-exposure prophylaxis for HIV and sexually transmitted infections to reduce the incidence among sexual and gender-based violence and rape survivors.
